# The folding of 5′-UTR human G-quadruplexes possessing a long central loop

**DOI:** 10.1261/rna.044578.114

**Published:** 2014-07

**Authors:** Rachel Jodoin, Lubos Bauer, Jean-Michel Garant, Abdelhamid Mahdi Laaref, Francis Phaneuf, Jean-Pierre Perreault

**Affiliations:** 1 Département de Biochimie, Faculté de Médecine et des Sciences de la Santé, Pavillon de Recherche Appliquée au Cancer, Université de Sherbrooke, Québec, Canada J1E 4K8

**Keywords:** G-quadruplex, RNA structure, 5′-UTR, in-line probing, translation regulation

## Abstract

G-quadruplexes are widespread four-stranded structures that are adopted by G-rich regions of both DNA and RNA and are involved in essential biological processes such as mRNA translation. They are formed by the stacking of two or more G-quartets that are linked together by three loops. Although the maximal loop length is usually fixed to 7 nt in most G-quadruplex-predicting software, it has already been demonstrated that artificial DNA G-quadruplexes containing two distal loops that are limited to 1 nt each and a central loop up to 30 nt long are likely to form in vitro. This report demonstrates that such structures possessing a long central loop are actually found in the 5′-UTRs of human mRNAs. Firstly, 1453 potential G-quadruplex-forming sequences (PG4s) were identified through a bioinformatic survey that searched for sequences respecting the requirement for two 1-nt long distal loops and a long central loop of 2–90 nt in length. Secondly, in vitro in-line probing experiments confirmed and characterized the folding of eight candidates possessing central loops of 10–70 nt long. Finally, the biological effect of several G-quadruplexes with a long central loop on mRNA expression was studied in cellulo using a luciferase gene reporter assay. Clearly, the actual definition of G-quadruplex-forming sequences is too conservative and must be expanded to include the long central loop. This greatly expands the number of expected PG4s in the transcriptome. Consideration of these new candidates might aid in elucidating the potentially important biological implications of the G-quadruplex structure.

## Abstract (precis)

This report demonstrates that G-quadruplexes with a long central loop are actually found in the 5′-UTRs of human mRNAs. Consideration of these new candidates might aid in elucidating the potentially important biological implications of the G-quadruplex structure.

## INTRODUCTION

Guanine-rich nucleic acid sequences can fold into a well-known tetrahelical structure called G-quadruplex. The basic building blocks of the G-quadruplex core are two or more G-quartets, which are planar arrangements of four guanines held together by Hoogsteen hydrogen bonds pairing (Gellert et al. 1962). The structure is formed by the stacking of the G-quartets on top of each other and is further stabilized by the binding of monovalent ions, especially Na^+^ and K^+^. A typical intramolecular G-quadruplex-forming sequence is composed of four tracks of two or more consecutive guanines (G-tracks) which are interspersed by three loops of variable lengths and nucleotide compositions. The stability of the structure is affected by several features, including the number of G-quartets, the possibility of bulge formation, the type and concentration of monovalent cations in solution, the sequence of the nucleic acid molecule itself, and the length of the loops composing the G-quadruplex (Burge et al. 2006; Mukundan and Phan 2013). Several studies focused on the bioinformatic analysis of G-quadruplexes in the human genome confirmed the presence of a significant number of potential G-quadruplex-forming sequences (PG4s) in various biologically relevant regulatory regions such as the promoter elements of genes, telomeres, and the UTRs of mRNAs (Huppert and Balasubramanian 2005; Eddy and Maizels 2006; Huppert et al. 2008; Beaudoin and Perreault 2010, 2013). The existence of RNA G-quadruplexes in human cells was recently confirmed using a structure-specific antibody (Biffi et al. 2013). A significant number of studies have linked G-quadruplexes to important biological processes, including mRNA splicing, polyadenylation, translation repression, and localization (Shafer and Smirnov 2000; Beaudoin and Perreault 2010, 2013; Marcel et al. 2011; Bugaut and Balasubramanian 2012; Millevoi et al. 2012), thus rendering them interesting potential therapeutic targets (Patel et al. 2007; Collie and Parkinson 2011; Marcel et al. 2011; McLuckie et al. 2013).

Biophysical studies have confirmed that RNA G-quadruplexes are generally thermodynamically more stable than their DNA counterparts (Sacca et al. 2005; Joachimi et al. 2009; Halder and Hartig 2011; Zhang et al. 2011). Moreover, RNA G-quadruplexes are restricted to adopting a parallel configuration caused by the stronger preference for an anti*-*conformation of the glycosidic bond between the ribose and guanine moieties (Halder and Hartig 2011). Considerable effort has been spent trying to understand the principles which govern the folding of G-quadruplexes (Hardin et al. 2000; Xue et al. 2011; Karsisiotis et al. 2013). Numerous articles have explored the contributions of the composition and length of the loops on the formation and topology of both DNA and RNA G-quadruplexes (Hazel et al. 2004; Risitano and Fox 2004; Rachwal et al. 2007; Guedin et al. 2009, 2010; Olsen et al. 2009; Zhang et al. 2011; Koirala et al. 2013; Kwok et al. 2013; Pandey et al. 2013), and some general conclusions regarding the loops have emerged. Firstly, in contrast to DNA, the topology of RNA G-quadruplexes is always parallel and independent of the loop length and sequence (Zhang et al. 2011; Pandey et al. 2013). Secondly, the stability of both DNA and RNA quadruplexes and the length of the loops are inversely related. In other words, G-quadruplexes with shorter loops exhibit higher stability than those with longer loops. However, it is very important to note that this holds true only for sequences with shorter loops. If a G-quadruplex structure harbors longer loops (>20 nt) a plateau is attained and the stability becomes less dependent on the loop length (Guedin et al. 2010; Pandey et al. 2013). Moreover, it was established that, if a very long central loop is accompanied by two short loops comprised of a single nucleotide each, the stability of the G-quadruplex was still relatively high, exceeding the physiological temperature (Guedin et al. 2010; Pandey et al. 2013). The majority of these studies were conducted on artificial DNA sequences in which the length of the loops did not exceed 30 nt. Despite the numerous studies, the issue of longer loops occurring in natural RNA G-quadruplexes still remains poorly explored. In accordance with these conclusions, it seemed plausible that 5′-UTR RNA G-quadruplexes with longer loop lengths could be stable enough to be formed and retrieved in the human transcriptome. If this is indeed the case, they could act as translational repressors (Beaudoin and Perreault 2010; Bugaut and Balasubramanian 2012). To verify these assumptions, a database of 1453 human 5′-UTR PG4s composed of two distal loops of length of 1 nt and a central loop of varying lengths, ranging from 2 to 90 nt, was constructed. The folding of eight representative PG4s with different central loop lengths was confirmed in vitro, and in some cases in cellulo. All of the PG4s investigated defy the classical algorithm respecting 7-nt-long loops only.

## RESULTS

### Database of G-quadruplexes possessing a long central loop

**FIGURE 1.**
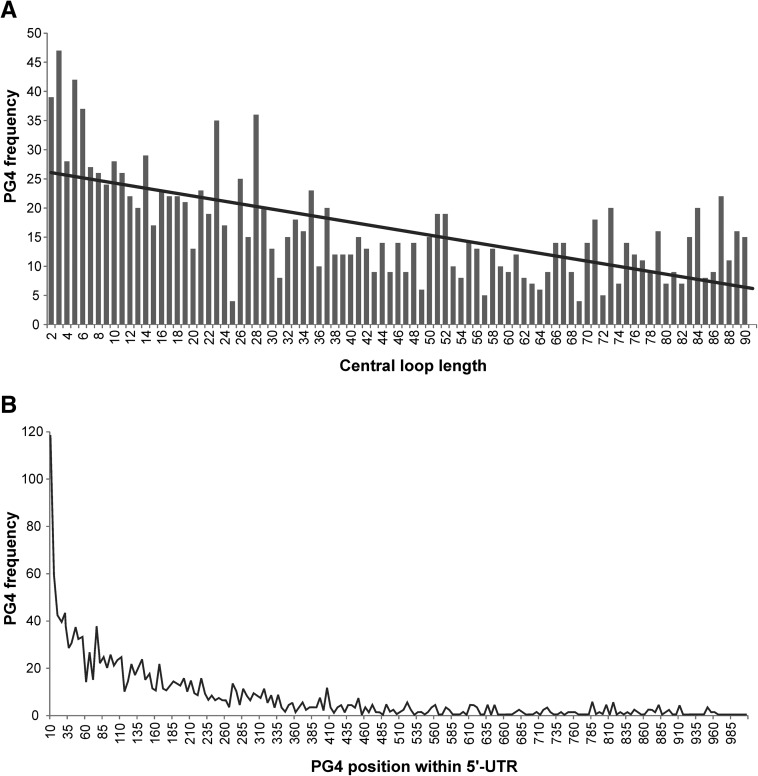
(*A*) Incidence of potential G-quadruplexes (PG4s) possessing central loops of varying lengths in a human 5′-UTR database. (*B*) Position of PG4s within the 5′-UTR.

Initially, PG4s were searched using the algorithm Gx–N_1_–Gx–N_2–90_–Gx–N_1_–Gx, where G stands for guanine, N for any nucleotide (A, U, C, and G), and *x* ≥ 3. This in silico analysis of the human 5′-UTRs yielded 1453 PG4 sequences with central loops ranging from 2 to 90 nt accompanied by 1-nt-long distal loops. Out of the 1453 PG4s, 1232 were comprised of central loops >8 nt, therefore deviating from the widely used search algorithm. The analysis of the constructed database permitted the observation of some interesting tendencies of the PG4 sequences found in human 5′-UTRs. Comparing the lengths of the central loop revealed that PG4s with shorter loops were more frequent (Fig. 1A) and that there was a tendency showing that the longer the loop, the fewer the number of PG4s retrieved. The positions of the PG4 within the 5′-UTR demonstrated that they tend to localize at the 5′-extremity of the 5′-UTR (Fig. 1B), which is in agreement with the work on RNA G-quadruplexes corresponding to the canonical definition (Huppert et al. 2008).

### In vitro folding of potential G-quadruplex-forming sequences possessing a long central loop

Representative, natural 5′-UTR PG4 sequences with variable central loop lengths were chosen from the database (Table 1) and subjected to in-line probing experiments to verify their ability to fold into G-quadruplex structures in vitro. This technique has been very successfully used to follow the formation of G-quadruplexes located in both 5′- and 3′-UTRs of RNA transcripts (Beaudoin and Perreault 2010, 2013). In addition, a step-by-step methodology of the whole in-line probing protocol, including the design of the PG4s, performing of the experiments, and the evaluation of the data, has already been described in detail (Beaudoin et al. 2013a). Briefly, this assay makes use of the natural instability of RNA to elucidate secondary structure characteristics. For instance, when a PG4 sequence adopts an intramolecular G-quadruplex structure, the nucleotides in the loops should bulge out of the RNA's structure and should therefore be more susceptible to spontaneous non-enzymatic cleavage of their phosphodiester bonds, a process that is favored by the presence of magnesium ions. To render the analysis more biologically relevant, extra 15-nt sequences were added to both ends of the PG4 sequence. This permitted observation of the formation of the G-quadruplex structure in its broader genomic context. In addition to the wild-type (wt) PG4 version, a mutated version in which some key guanines were substituted for adenines (G/A-mut) was synthesized in each case. The G/A-mutant served as a negative control for G-quadruplex formation as it possessed only minor changes in its RNA sequence compared with that of the wt. Knowing that Li^+^ cations are unable to stabilize the G-quadruplex structure, due to their small size, another layer of control was added and the in-line reactions were performed in the presence of 100 mM of both K^+^ and Li^+^ to favor and disfavor, respectively, the formation of G-quadruplexes.

**TABLE 1.**
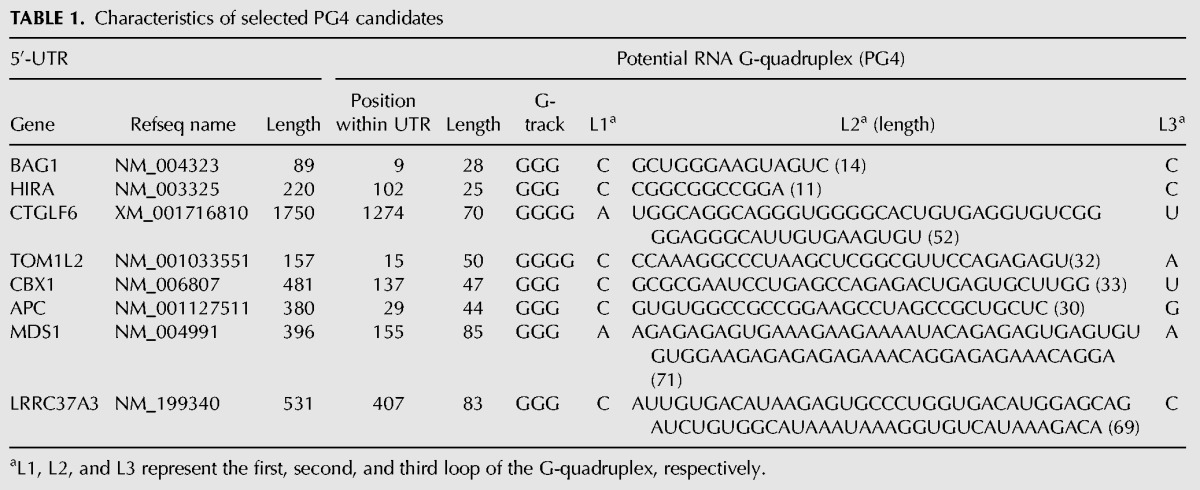
Characteristics of selected PG4 candidates

#### BAG1

**FIGURE 2.**
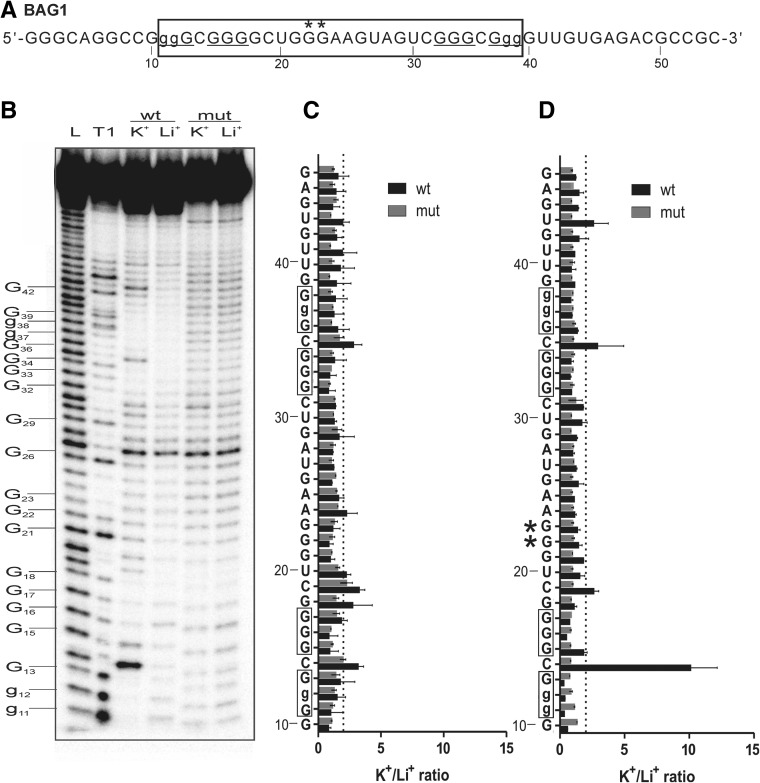
In-line probing results of the BAG1 PG4 candidate which possesses a 14-nt-long central loop. (*A*) Nucleotide sequence of the characterized BAG1 wt transcript. The lowercase guanines (g) correspond to those substituted for adenines in the G/A-mutant versions. Guanines mutated in the central loop are denoted by asterisks (*). Underlined G-tracks indicate the nucleotides predicted to be involved in the G-quadruplex formation. The boxed sequence denotes the predicted PG4. (*B*) Autoradiogram of a 10% denaturing (8 M urea) polyacrylamide gel of the in-line probing of both the 5′-labelled BAG1 wt and G/A-mutant PG4 versions performed in the presence of 100 mM of either LiCl or KCl. Lanes *L* and *T1* indicate the alkaline hydrolysis and ribonuclease T1 mapping lanes, respectively. The positions of the guanines are indicated on the *left* of the gel. The lowercase guanines were converted to adenines in the mutant version. (*C*,*D*) K^+^/Li^+^ ratios of the band intensities of the BAG1 wt and the G/A-mutant for each nucleotide. The K^+^/Li^+^ ratios are shown in dark gray for BAG1 wt and in light gray for the BAG1 G/A-mutant. The boxed guanines represent the predicted G-tracks. The dotted line represents the twofold threshold that denotes a significant gain in flexibility. The sequence is indicated on the *y*-axis. The lowercase Gs shown on the *y*-axis are mutated to As in the mutant version. The asterisk (*) indicates guanines mutated to adenines in the central loop. Each bar represents the average of two independent experiments, and the error bars represent the standard deviations.

Initially the PG4 found in the 5′-UTR of the human BAG1 mRNA was chosen from the database to assess its ability to form a G-quadruplex possessing a long central loop. The BAG1 PG4 was predicted to be comprised of 28 nt with a central loop of 14 nt and forming a G-quadruplex with three G-quartet layers (Table 1). The analyzed sequence of BAG1 is shown in Figure 2A. The boxed nucleotides represent the PG4, and the tracks of guanines predicted to be involved in the formation of the G-quadruplex are underlined. A typical autoradiogram for an in-line probing analysis of both the wt and G/A-mutant versions of the BAG1 PG4 is illustrated in Figure 2B. Differences in the intensities of some bands were observed at several positions of the wt PG4 in the presence of 100 mM KCl as compared in the presence of 100 mM LiCl. More specifically, the bands corresponding to the nucleotides found in the predicted loops that are located between the guanine tracks (i.e., C14, G18, C19, U20, A24, and C35) became more intense only for the wt version in the presence of KCl. In addition, the inability of the G/A-mutants to fold into a G-quadruplex structure was confirmed, regardless of the type of the cation used. To quantitatively evaluate the in-line probing analysis, the intensity of each band in the K^+^ lane was divided by that of the corresponding band in the Li^+^ lane. The retrieved K^+^/Li^+^ ratios for each band were further used to create bar graphs (Fig. 2C) with the nucleotide sequence plotted on the *y*-axis and the intensity ratios on the *x*-axis. A nucleotide was considered significantly more accessible when this ratio was higher than an arbitrarily fixed threshold of 2. As expected, the ratios of the nucleotides located between the tracks of guanines were superior to the arbitrary threshold, suggesting that BAG1 forms a RNA G-quadruplex with a 14-nt-long central loop. Since the sequence of the central loop contains an additional G-track, it is reasonable to assume that it might be involved in the formation of alternative G-quadruplexes. The extra G-track could provide multiple folding scenarios and support the formation of various G-quadruplex structures. In this case the resulting cleavage pattern would reflect the sum of multiple G-quadruplex species present in solution during the 40-h-long incubation procedure. To get insight into this hypothesis, a mutant BAG1 was constructed. Guanines G22 and G23, which are located in the central loop, were changed to adenines. The in-line probing was performed on this mutated sequence, followed by a quantitative analysis of the bands. The significant increase in the intensity of nucleotide C14 located between the first and second G-track implies that a new equilibrium was established and that only one species with a long central loop was favored (Fig. 2D).

#### HIRA

**FIGURE 3.**
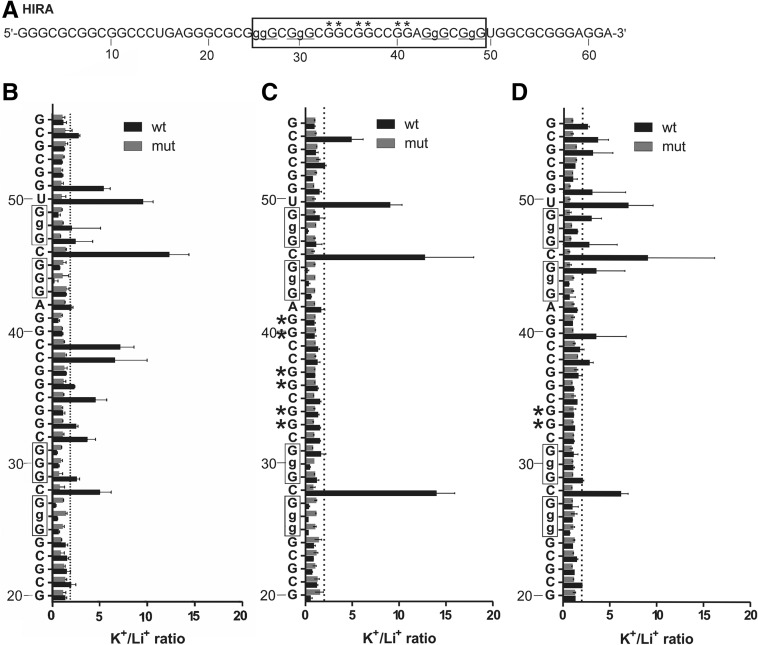
In-line probing results of the HIRA PG4 candidate which possesses an 11-nt central loop. (*A*) Nucleotide sequence of the characterized HIRA wt transcript. The lowercase guanines (g) correspond to those substituted for adenines in the G/A-mutant version. Guanines mutated in the central loop are denoted by asterisks (*). Underlined G-tracks indicate the nucleotides predicted to be involved in the G-quadruplex formation. The boxed sequence denotes the predicted PG4. (*B*–*D*) K^+^/Li^+^ ratios of the band intensities of the HIRA wt and the G/A-mutant in vitro G-quadruplex version for each nucleotide. The K^+^/Li^+^ ratios are shown in dark gray for the HIRA wt and in light gray for the HIRA G/A-mutant. The boxed guanines represent the predicted G-tracks. The lowercase Gs shown on the *y*-axis are mutated to As in the mutant version. The dotted line represents the twofold threshold that denotes a significant gain in flexibility. The nucleotide sequence is indicated on the *y*-axis. The asterisk (*) indicates guanines mutated to adenines in the central loop. Each bar represents the average of two independent experiments, and the error bars represent the standard deviations.

Based on observations made on BAG1 and the guanine track located in the central loop, further investigation focused on HIRA (Table 1), a candidate predicted to fold into a G-quadruplex structure composed of three G-quartets with an 11-nt-long central loop harboring three guanine doublets (identified with asterisks in Fig. 3A). In the presence of KCl the wt sequence displayed an in-line cleavage pattern typical of the formation of multiple G-quadruplex species. Besides the nucleotides which were predicted to be the first and third single nucleotide loops of the PG4 (C28, C46), additional accessible sites superior to the arbitrary threshold were identified in the long central loop (C32, G33, C35, G36, C38, and C39). The localization of residues between doublets of guanines (C32, C35, C38, and C39) strongly supports the existence of an alternative G-quadruplex consisting of two G-quartets, the minimum requirement for the structure. Because of the presence of multiple G-tracks, and of several folding combinations, it is complicated to evaluate which of them are involved in the formation of particular G-quadruplexes. Moreover, a guanine doublet located just after the predicted PG4 might be also involved in the formation of an alternative G-quadruplex as evidenced by the superior accessibility of nucleotide U50. To prove the existence of an alternative G-quadruplex topology composed of two G-quartets, G to A mutations were introduced into the three guanine doublets. The cleavage susceptibility of the nucleotides located between the doublets did indeed decrease under the threshold, and the folding of the originally predicted PG4 with a long central loop was promoted, as is indicated by the increased cleavage ratios of both C28 and C46 (Fig. 3C). The mutation of only the first guanine doublet reduced the in-line cleavage susceptibility of nucleotides C32 and C35 (Fig. 3D). This result is in concordance with the previous mutation of all guanine doublets, and reinforces the hypothesis that the doublets offer alternative folding pathways.

#### CTGLF6

**FIGURE 4.**
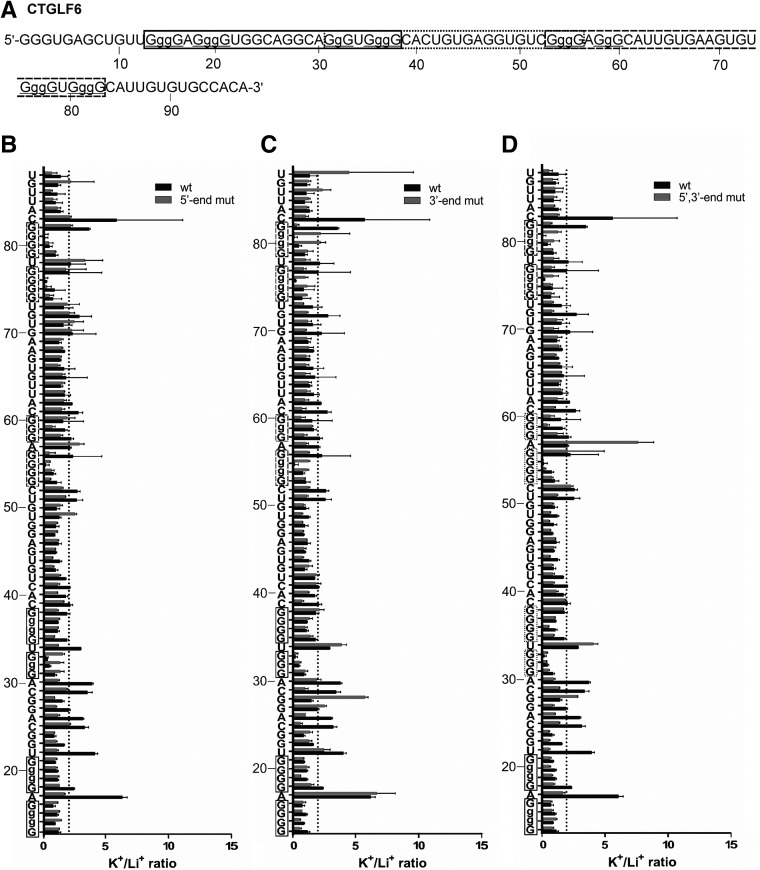
In-line probing results of the CTGLF6 PG4 candidate showing three overlapping PG4s, possessing a 10-, 16-, or 14-nt central loops. (*A*) Nucleotide sequence of the characterized CTGLF6 wt transcript. The lowercase guanines (g) correspond to those substituted for adenines in the G/A-mutant version. Underlined G-tracks indicate the predicted nucleotides involved in the G-quadruplex formation. The boxed sequences in different frames denote the predicted PG4s. (*B*–*D*) K^+^/Li^+^ ratios of the band intensities of the CTGLF6 wt and the different G/A-mutants in vitro G-quadruplex versions for each nucleotide. (*B*) CTGLF6 wt and 5′-end G/A-mutant, (*C*) CTGLF6 wt and 3′-end G/A-mutant, and (*D*) CTGLF6 wt and 5′-, 3′-end G/A-mutant. The K^+^/Li^+^ ratios are shown in dark gray for the CTGLF6 wt and in light gray for the different CTGLF6 G/A-mutants. The boxed guanines represent the predicted G-tracks. The dotted line represents the twofold threshold that denotes a significant gain in flexibility. The nucleotide sequence is indicated on the *y*-axis. The lowercase Gs shown on the *y*-axis are mutated to As in the mutant version. Each bar represents the average of two independent experiments, and the error bars represent the standard deviations.

The next candidate to be examined was CTGLF6, a sequence capable of folding into multiple G-quadruplex species with different loop lengths depending on which combination of G-tracks is considered to be involved in the formation of a particular structure (Table 1). The predicted PG4s harboring 10-, 16-, and 14-nt-long central loops are denoted in solid, dotted, and dashed boxes, respectively (Fig. 4A). In addition, the PG4 consisting of the first two G-tracks of the first PG4 (solid box) and the last two G-tracks of the third PG4 (dashed box) with a long 52-nt central loop must also be considered. Furthermore, the existence of G-quadruplex subunits arranged in tandem between the first (solid) and third (dashed) PG4 intercalated with the central loop of the second PG4 (dotted) also seems to be a plausible possibility. Several mutations were performed to modulate the folding toward one specific structure by impairing the participation of specific G-tracks in the formation of G-quartets. The in-line probing pattern of the wt sequence in the presence of K^+^ corresponds to the formation of two consecutively arranged G-quadruplexes, as expected (PG4 in the solid and dashed boxes, respectively). However, it is important to note that the accessibility of the nucleotides located between the G-tracks of the G-quadruplex located at the 3′-end is on the edge of the arbitrarily defined threshold. The first series of mutations was introduced with the intention of impairing the formation of the G-quadruplex located in the 5′-end. A decrease in the cleavage ratios for nucleotides A17 and U34 in the single-stranded loops was observed. On the other hand, the intensity ratios of residues A57 and U78, situated amid the G-tracks of the 3′-end G-quadruplex, were slightly increased, indicating the promotion of this structure (Fig. 4B). The same kind of behavior, but in an inverted order, was observed when the G-quadruplex situated at the 3′-end was mutated so as to abolish its formation. Specifically, the decreased cleavage ratios of nucleotides A57 and U78 in the first and third loops of the 3′-end G-quadruplex was accompanied by an increase in the cleavage ratios of the nucleotides located in the short loops (nucleotides A17 and U34) of the 5′-end G-quadruplex (Fig. 4C). The last mutation performed was designed to promote the formation of a G-quadruplex structure in the center of the sequence (PG4 in the dotted box) by impairing the first two guanine tracks in the 5′-end PG4 (solid box) and the last two G-tracks in the 3′-end PG4 (dashed box). The resulting structure confirmed expectations, as both the first (U34) and third loops (A57) exhibited higher cleavage ratios as compared with the wt sequence (Fig. 4D).

#### TOM1L2, CBX1, and APC

**FIGURE 5.**
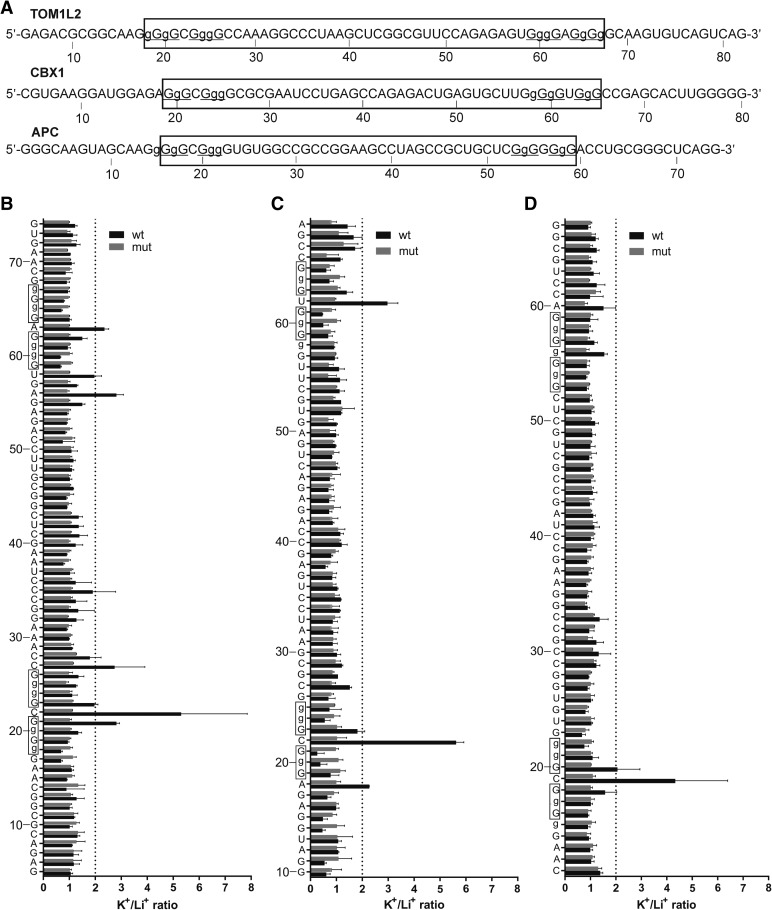
In-line probing results of the TOM1L2, CBX1, and APC, PG4s possessing central loops of 32, 33, and 30 nt, respectively. (*A*) Nucleotide sequences of the characterized wt transcripts. The lowercase guanines (g) correspond to those substituted for adenines in the G/A-mutant version. Underlined G-tracks indicate the nucleotides predicted to be involved in the G-quadruplex formation. The boxed sequences denote the predicted PG4. (*B*–*D*) K^+^/Li^+^ ratios of band intensities of the wt and G/A-mutant for each nucleotide. (*B*) TOM1L2, (*C*) CBX1, and (*D*) APC. The K^+^/Li^+^ ratios are shown in dark gray for the wt and in light gray for the G/A-mutant. The boxed guanines represent the predicted G-tracks. The dotted line represents the twofold threshold that denotes a significant gain in flexibility. The nucleotide sequence is indicated on the *y*-axis. The lowercase Gs shown on the *y*-axis are mutated to As in the mutant version. Each bar represents the average of two independent experiments, and the error bars represent the standard deviations.

Three candidates, namely TOM1L2, CBX1, and APC containing 32-, 33-, and 30-nt-long central loops, respectively, were examined to confirm their ability to fold into RNA G-quadruplex structures (Table 1). With the exception of APC, the wt candidates displayed the typical banding patterns corresponding to the formation of G-quadruplexes in the presence of KCl. As expected, the superior cleavage ratios of nucleotides C22 and A63 in case of TOM1L2 and C22 and U62 in case of CBX1, which are located between the G-tracks, confirmed the initial observations (Fig. 5A,B,C). The increased cleavage ratio at position G21 of TOM1L2 suggests that this nucleotide ends up in the loop with C22. This indicates that a G-quadruplex with a first loop of 2 nt is formed (Fig. 5B). In the case of APC, the inferior cleavage ratio of G56, which is located between the last two G-tracks, did not support the conclusion that a G-quadruplex is formed by this PG4 (Fig. 5D). None of the three PG4s described contained extra G-tracks located in the central loop, nor in the 15-nt-long overhangs flanking both sides of the predicted PG4s. This feature simplifies the evaluation and the interpretation of the data due to the absence of multiple G-quadruplex species.

#### MDS1 and LRRC37A3

**FIGURE 6.**
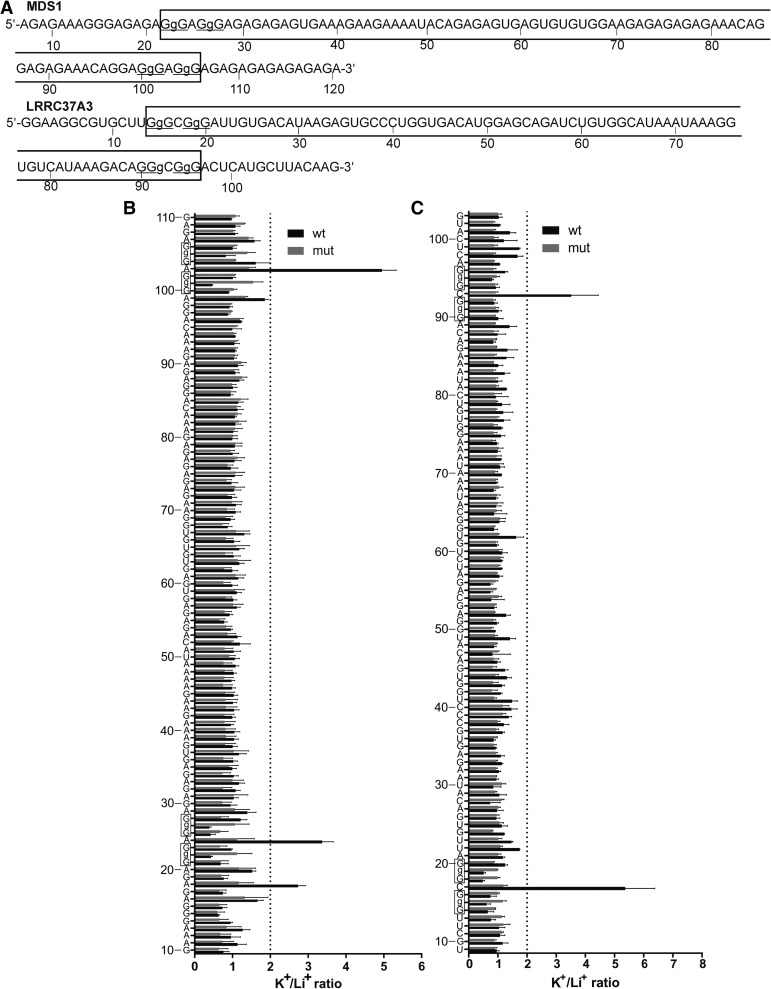
In-line probing results of the MDS1 and LRRC37A3 possessing central loops of 71 and 69 nt, respectively. (*A*) Nucleotide sequences of the characterized wt transcripts. The lowercase guanines (g) correspond to those substituted for adenines in the G/A-mutant version. The underlined G-tracks indicate the nucleotides predicted to be involved in the G-quadruplex formation. The boxed sequences denote the predicted PG4. (*B*,*C*) K^+^/Li^+^ ratios of band intensities of the wt and the G/A-mutant for each nucleotide. (*B*) MDS1, (*C*) LRRC37A3. The K^+^/Li^+^ ratios are shown in dark gray for the wt and in light gray for the G/A-mutant. The boxed guanines represent the predicted G-tracks. The dotted line represents the twofold threshold that denotes a significant gain in flexibility. The nucleotide sequence is indicated on the *y*-axis. The lowercase Gs shown on the *y*-axis are mutated to As in the mutant version. Each bar represents the average of two independent experiments, and the error bars represent the standard deviations.

Lastly, two PG4s with central loops composed of 71 (MDS1) and 69 (LRRC37A3) nt (Table 1) were analyzed by in-line probing. In both cases, the wt sequences displayed exclusive higher K^+^/Li^+^ cleavage ratios of the residues predicted to be found in the single-stranded loops located between the G-tracks. More precisely, these residues correspond to nucleotides A24 and A103 for MDS1 and C17 and C93 for LRRC37A3. In comparison, the G/A-mutants did not pose such characteristics, regardless of whether incubation was performed in the presence of LiCl or KCl (Fig. 6). The assumption in the lack of cleavage difference for the nucleotides of the central loop could be explained by the presence of the same structure in both G-quadruplex favorable and unfavorable conditions. This argument is further supported by SHAPE probing experiments of the nucleotides located in the long central loop (data not shown). The SHAPE banding patterns obtained for the wt and G/A-mutant constructs were identical, indicating highly similar if not identical structures. The exceptionally long central loop of these PG4s sets a new limit of what might be still considered as an in vitro G-quadruplex-forming sequence.

### In cellulo folding of G-quadruplexes possessing a long central loop

**FIGURE 7.**
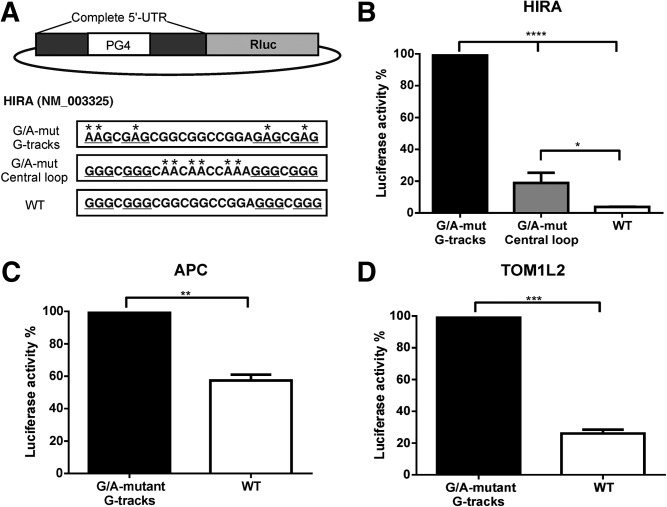
Effect of a G-quadruplex possessing a long central loop on luciferase activity. (*A*) Schematic representation of the vector construction with the different sequences used for the HIRA candidate constructs. The PG4 region is boxed, the guanines involved in the G-tracks are underlined, and nucleotides identified with an asterisk are the guanines that were mutated to adenines in the different G/A-mutant constructs. Values were first normalized by dividing the value of Rluc by the value of the control Firefly luciferase (Fluc). The percentage (%) of luciferase activity was set to 100% for all of the G-tracks G/A-mutant constructs. The luciferase activity values of the other construct were divided by the value of their corresponding G-tracks G/A-mutant construct and then multiplied by 100. (*B*) Luciferase activity of the different HIRA constructs each with a central loop of 11 nt. (*C*) APC possessing a central loop of 30 nt, and (*D*) TOM1L2 possessing a central loop of 32 nt. For these three examples, the wt constructs which can fold into a G-quadruplex reduced the luciferase activity. The results are the means of at least two independent experiments, and the error bars represent the standard deviations. *P*-value were calculated by unpaired Student *t*-test. (*) *P* < 0.05, (**) *P* < 0.01, (***) *P* < 0.001, (****) *P* < 0.0001.

Encouraged by the results indicating that these G-quadruplex structures were folded in vitro, the next step was to verify their biological relevance by investigating their folding in cellulo. Multiple RNA G-quadruplex motifs located in the 5′-UTR of genes are reported to inhibit translation (Millevoi et al. 2012). With the use of dual-luciferase reporter assays, we investigated whether or not some of the above candidates possessing unusual long central loops of 11, 30, and 70 nt could trigger the same effect. The complete (full-length) 5′-UTRs of the candidates were inserted upstream of the Renilla luciferase (Rluc) reporter gene. The levels of Rluc expression, normalized over the control Firefly luciferase (Fluc) expression, were compared between the wt constructs and the different G/A-mutants. The mutations used were the same as for the in vitro in-line probing assays. Figure 7A presents a schema of the different constructs of the HIRA candidate. To facilitate the comparison between each construction, and between different candidates, the luciferase activity of each construct was normalized over its corresponding G-tracks G/A-mutant and reported as a percentage. As expected for G-quadruplex formation, luciferase activity of the HIRA wt construct was reduced almost 90% as compared with the G/A-mutant construct which cannot adopt a G-quadruplex (Fig. 7B). A smaller, but still important decrease of ∼80% was also observed for the construction with G/A-mutation in the central loop. Accordingly to the in vitro in-line probing results, the HIRA wt construct could adopt multiple G-quadruplexes depending on the different combinations of the G-tracks used to form the structure. It seems that this pool of variable G-quadruplexes with different loop lengths and G-tracks has a higher detrimental impact on the expression of the luciferase gene than does a pool where a G-quadruplex with a long central loop is dominant, as is the case for the central loop G/A-mutant construct. However, in both cases, G-quadruplexes were folded in cellulo. Similar results were observed for both the APC and the TOM1L2 candidates, which possess central loops of 30 and 32 nt, respectively. Decreases in the luciferase activities of ∼40% for APC and of ∼75% for TOM1L2, due to G-quadruplex formation, were observed (Fig. 7C,D). Data obtained from in cellulo experiments with APC are in disagreement with the in vitro results, which did not unambiguously confirm the formation of a G-quadruplex. The down-regulation of luciferase expression via the presence of the 5′-UTR sequence of APC upstream of the luciferase reporter gene was confirmed (Fig. 7C). The likely reasons for this difference could be the following: (i) The single nucleotide loop of G56 (Fig. 5D) is very well protected from cleavage; and (ii) the conditions found in the cell, specifically crowding or the presence of G-quadruplex binding proteins, might provide further stabilization of the G-quadruplex. Differences between in vitro and in cellulo results were also observed for the candidates with central loops of >69 nt (MDS1 and LRRC37A3). Even though in-line probing results showed patterns of G-quadruplex formation, no difference in luciferase activity was measured between the wt and G-tracks G/A-mutant constructs (data not shown), indicating either that cellular conditions are not favorable for the formation of G-quadruplexes with such long central loops or that they are not stable enough to affect translation significantly. In conclusion, the observed decreases in luciferase activity demonstrated that G-quadruplexes that include a long central loop up to 30 nt in length present inside the 5′-UTR are stable enough to negatively impact an essential biological process, in this case mRNA translation.

## DISCUSSION

The results presented above confirm that potential RNA G-quadruplex-forming sequences located in human 5′-UTRs harboring a long central loop (2–90 nt) and two single nucleotide distal loops are relatively common and might be physiologically relevant. Both in vitro and in cellulo data are in agreement with the earlier work of others (Guedin et al. 2010; Bugaut and Balasubramanian 2012; Pandey et al. 2013) and question the legitimacy of the very strict G-quadruplex search algorithm that has been used in many studies, an algorithm which considers loops only up to 7 nt long. Although this report changes the frontier of what might still be considered as putative G-quadruplex-forming sequence, the approach used did not permit the elucidation of the upper limit of loop length consistent with G-quadruplex formation. The comprehensive bioinformatic search reported here has identified 1453 5′-UTR PG4 sequences possessing a long central loop located on the complementary strand. In comparison, a similar study published earlier by our laboratory using the above-mentioned overly strict search algorithm limited to loops consisting of maximum 7 nt identified 7198 PG4 sequences located on the complementary strand (Beaudoin and Perreault 2010). If only PG4s with a central loop of ≥8 nt in length are considered, 1232 additional PG4s possessing a long central loop went unnoticed by the previous limited search. This number represents a 17.11% increase in newly identified PG4s. It is likely that additional searches for PG4s harboring either a long first or third loop accompanied by two single nucleotide long loops would further increase the number of otherwise unidentified PG4s. However, thermodynamic data from previous biophysical studies carried out on artificial DNA sequences do not support the folding of G-quadruplexes with such loop arrangements (Guedin et al. 2010). It is proper to note that bioinformatical approaches usually overestimate the actual number of G-quadruplexes present in the cell since they are restricted to sequence criteria only. Moreover, an analysis performed with a recently published scoring system used to identify RNA G-quadruplex folding (Beaudoin et al. 2013b) suggests that 40% of the PG4 candidates identified in silico are prone to fold into G-quadruplex structures based on a predicting value respecting both the ratio of consecutive guanines and the cytosine enrichment (data not shown).

This report investigated eight G-quadruplexes including long central loop. All of these RNA species were folded into G-quadruplex in vitro with the exception of APC, but only three out of the five tested in cellulo repressed translation, suggesting that less were formed in the cell. It is important to mention that the PG4 sequences that were chosen seemed to possess a high probability of folding into a G-quadruplex. For example, all of the selected candidates seemed to lack a Watson-Crick base-pair-based secondary structure stable enough to compete against the G-quadruplex structure. This was perhaps the most important criteria used, as the goal was to unambiguously demonstrate that some PG4 possessing a long central loop were effectively folded. It is clear that, among the 5′-UTR PG4 sequences retrieved, there is a proportion of these sequences that does not fold into the G-quadruplex structure.

G-quadruplexes are known to be topologically extremely variable, and the folding of the structure is often driven by more complicated pathways which do not necessarily respect a simple two-state equilibrium model between the folded and unfolded state, as was recently demonstrated for the human telomeric DNA sequence (Bian et al. 2014). The final topology of the structure is usually influenced by a combination of different intrinsic and extrinsic factors, including the sequence of the molecule itself, the natures and concentrations of any monovalent ions, molecular crowding, the pH, and the temperature, among others. Unlike artificially designed sequences, which were primarily used in various biophysical studies in order to avoid the formation of unwanted folding possibilities, PG4s within biologically relevant regulatory regions such as the UTRs are very diverse in terms of G-tracks and loop lengths. This feature determines the variability in the number of stacked G-quartets and connecting loops. The presence of multiple G-quadruplex species in solution is one of the major problems complicating data evaluation in many biophysical approaches, including circular dichroism, NMR, and UV melting, where the resulting data often represent a mixture of different DNA G-quadruplex structures (Viglasky et al. 2010). This report demonstrates that additional G-tracks located either in the loops or the regions flanking the predicted PG4s readily fold into a mixture of different G-quadruplex structures (see the candidates BAG1, HIRA, and CTGLF6) (Figs. 2–4). In light of this finding, in-line probing appears to be the method of choice for assessing the complexity of all of the folding possibilities, which are then further reinforced by structural information. Among other advantages of in-line probing, the requirement of only trace amounts of RNA (<1 nM), which should favor intramolecular folding, and the ability to study short as well as long RNA molecules under different salt conditions should be stressed. It is noteworthy that the folding of central loop sequences exceeding the length of 8 nt performed by RNAfold (Lorenz et al. 2011) revealed that the vast majority of the sequences adopt a stem–loop secondary structure (data not shown). The coexistence of multiple G-quadruplex species, the exceptional length of some PG4s, and the very likely presence of an alternative structure in the central loop represents a limiting factor for well-established techniques such as circular dichroism and UV melting. To avoid the limitations and data misinterpretation of the in vitro experiments, the folding of some selected candidates was verified in cellulo by cloning the entire 5′-UTR containing the PG4 of interest upstream of the luciferase reporter gene. This approach successfully demonstrated the down-regulation of luciferase expression for the wt sequences when compared with the mutated one for candidates with 11-, 30-, and 32-nt-long central loops. This strongly implies that G-quadruplexes with long loops might be stable enough to regulate gene expression on a cellular level.

This work demonstrates that it is possible to find G-quadruplexes possessing a long central loop in human 5′-UTRs. In addition, the folding of some interesting candidates possessing a central loop varying in length from 11 to 71 nt in vitro and 11 to 32 nt in cellulo has been confirmed. It is noteworthy that the presence of any extra G-tracks in the central loop provides additional folding pathways, resulting in the presence of multiple G-quadruplex species. The introduction of mutations that abolish the participation of these extra G-tracks in the central loop seems to be an effective way of regulating the folding of G-quadruplexes. The increased in-line cleavage of the nucleotides amid the guanine doublets in the central loop of the HIRA candidate indicates that G-quadruplexes with only two G-quartet layers might be in competition with the more stable one consisting of three G-quartets located within the same RNA molecule. The case of CTGLF6 provides proof that two G-quadruplexes arranged in tandem within one RNA molecule might coexist at the same time, and that the mutations can promote the folding of a particular structure. The in vitro folding of MDS1 and LRRC37A3, both of which possess exceptionally long central loops of 71 and 69 nt, respectively, defies the widely accepted definition of a G-quadruplex and calls for a revision of the previously established algorithm that considers only 7-nt-long loops. The existence of G-quadruplexes possessing long loops provides additional targets for drug design and new sites for protein–G-quadruplex interactions.

## MATERIALS AND METHODS

### Bioinformatics

The potential human G-quadruplex sequences used in this study were chosen from a 5′-UTR database derived from UTRdb and Transterm (Mignone et al. 2005; Jacobs et al. 2009). PG4s were identified using the program RNAmotif (Macke et al. 2001) by describing an algorithm respecting the pattern Gx–N_1_–Gx–N_2–90_–Gx–N_1_–Gx, where G stands for a guanine and N for any nucleotide (A, U, C, and G). The retrieved sequences were further analyzed using home written Perl scripts, and were manually cured to obtain the database of PG4s possessing a long central loop provided in the Supplemental Material as an Excel sheet.

### RNA synthesis

All sequences used in the in vitro experiments were synthesized by in vitro transcription using T7 RNA polymerase as described previously (Beaudoin and Perreault 2010). Two overlapping oligonucleotides (2 mM each, Invitrogen) were annealed and a double-stranded DNA was obtained by filling in the gaps using purified Pfu DNA polymerase in the presence of 5% dimethyl sulfoxide (DMSO, Fisher). The double-stranded DNA sequence was then ethanol-precipitated. The resulting DNA templates contained the T7 RNA promoter sequence followed by the PG4 sequence. Transcription reactions were performed in a final volume of 100 µL using purified T7 RNA polymerase in the presence of RNase OUT (20 units, Invitrogen), pyrophosphatase (0.01 units, Roche Diagnostics), and 5 mM NTP in a buffer containing 80 mM HEPES-KOH pH 7.5, 25 mM MgCl_2_, 2 mM spermidine, and 40 mM DTT. The reactions were incubated for 2 h at 37°C, at which point they were treated with DNase RQ1 (Promega) for 20 min at 37°C. The RNA was then purified by phenol:chloroform extraction followed by an ethanol precipitation. RNA was fractionated by denaturing 10% polyacrylamide gel electrophoresis (8 M urea) (PAGE; 19:1 acrylamide to bisacrylamide) using 45 mM Tris-borate pH 7.5, 1 mM EDTA solution as running buffer. After electrophoresis, the RNAs were visualized by UV shadowing and the bands corresponding to the correct size of the PG4s were excised from the gel and the transcripts eluted overnight at room temperature in buffer containing 1 mM EDTA, 0.1% SDS, and 0.5 M ammonium acetate. The PG4s sequences were then ethanol-precipitated, dried, and dissolved in water. The concentrations were determined by spectrometry at 260 nm using a NanoVue system (GE Healthcare).

### Radioactive 5′-end RNA labelling

In order to produce 5′-end-labeled RNA molecules, purified transcripts (50 pmol) were dephosphorylated at 37°C for 30 min by adding 5 units of antarctic phosphatase (New England BioLabs) in a final volume of 10 μL containing 50 mM Bis-propane pH 6.0, 1 mM MgCl_2_, 0.1 mM ZnCl_2_, and 20 units RNase OUT (Invitrogen). The enzyme was inactivated by incubation for 5 min at 65°C. The dephosphorylated RNAs (10 pmol) were 5′-end-radiolabeled using 7.5 units of T4 polynucleotide kinase (Promega) for 1 h at 37°C in the presence of 3.2 pmol of [γ-_32_P]ATP (6000 Ci/mmol; New England Nuclear). The reactions were stopped by the addition of two volumes of formamide dye buffer (95% formamide, 10 mM EDTA, 0.025% bromophenol blue, and 0.025% xylene cyanol). The RNAs molecules were purified by 10% polyacrylamide 8 M urea gel electrophoresis. The bands corresponding to the 5′-end-labeled RNAs were then detected by autoradiography and the portions of gel containing the correct sizes were excised and recovered as described in the RNA synthesis section. The eluted and precipitated 5′-end-labeled transcripts were dissolved in 20 μL ultrapure water, and the final radioactivity was calculated using a Cerenkov counter (Bioscan QC-2000).

### In-line probing experiment

Trace amounts of 5′-end-labeled RNA (50,000 cpm, <1 nM) were heated at 70°C for 5 min, and then slow-cooled to room temperature >1 h in buffer containing 20 mM lithium cacodylate pH 7.5 and 100 mM of either LiCl or KCl in a final volume of 10 μL. Thereafter, the final volume of each sample was adjusted to 20 μL such that the final concentrations were 30 mM lithium cacodylate pH 8.5, 20 mM MgCl_2_, and 150 mM of either LiCl or KCl. The reactions were then incubated for 40 h at room temperature, at which point the RNA was ethanol-precipitated in presence of glycogen and then RNAs dissolved in 20 µL of formamide loading buffer (95% formamide and 10 mM EDTA, 0.025% bromophenol blue). For the alkaline hydrolysis ladder, 50,000 cpm of 5′-end-labeled wt RNA (<1 nM) was dissolved in water in a final volume of 5 µL, 1 µL of NaOH was added, and the reaction incubated for 1 min at room temperature prior to being quenched by the addition of 3 µL of 1 M Tris-HCl pH 7.5. The RNA molecules were then ethanol-precipitated and dissolved in 20 µL of formamide loading buffer. For the RNAse T1 ladder, 50,000 cpm of 5′-end-labeled wt RNA (<1 nM) was dissolved in 9 µL of buffer containing 20 mM Tris-HCl pH 7.5, 10 mM MgCl_2_, and 100 mM LiCl. The mixture was incubated for 2 min at 37°C in the presence of 0.6 units of RNAse T1 (Roche Diagnostics), and then was quenched by the addition of 20 µL of formamide loading buffer. The radioactivities of both the in-line probing samples and the ladders were measured, using a Cerenkov counter (Bioscan QC-2000), and equal amounts in terms of counts per minute for all samples were fractionated on denaturing (8 M urea) 10% polyacrylamide gels. The resulting gels were dried and the bands visualized by exposing them to a phosphoscreen (GE Healthcare) and then analyzing it using a Typhoon Trio instrument (GE Healthcare).

### Data analysis

In-line probing gels were analyzed using the Semi-Automated Footprinting Analysis (SAFA) software (Das et al. 2005; Laederach et al. 2008). The RNase T1 ladder lane was used as the “anchor” line, using the guanines as cleavage sites for the sequence reference in SAFA. The raw intensities of each band under different salt conditions were determined and exported into a text file. The file was then opened with the Excel program in order to produce a usable table. Subsequently, the intensity of each band in the lanes representing the favorable conditions in the presence of KCl was divided by the intensity of the corresponding band in the LiCl lane (the unfavorable condition). Each in-line probing experiment was performed in duplicate. The averages and standard deviations were calculated for the K^+^/Li^+^ ratios for each nucleotide. These values were used to generate bar graphs, plotting the K^+^/Li^+^ ratio on the *x*-axis and the nucleotide sequence on the *y*-axis.

### In cellulo luciferase assay

The complete 5′-UTR sequences of the wt and various G/A-mutants of the HIRA, APC, and TOM1L2 candidates flanked by *Nhe*I restriction site was synthesized in vitro via multiple steps of PCR annealing and filling in of sets of overlapping oligonucleotides (Invitrogen). Complete 5′-UTR of both the wt and G/A-mutant constructs of MDS1 and LRRC37A3 flanked by *Nhe*1 restriction sites were obtained by custom gene synthesis (Biomatik). The list of the oligonucleotides and complete 5′-UTR sequences used are available in the Supplemental Material. The G/A mutations were the same as those in the in vitro constructs. The constructs were inserted upstream of the Renilla luciferase (Rluc) reporter gene in the *Nhe*1 restriction site of the pRL-TK vector (Promega) or the psiCHECK-2 vector for the HIRA constructs (Promega). All sequences were verified by DNA sequencing.

HEK 293 cells were cultured in Dulbecco's Modified Eagle Medium (DMEM, Wisent) supplemented with 10% foetal bovine serum (FBS, Wisent) and 1 mM sodium pyruvate (Wisent) at 37°C in a 5% CO_2_ and 100% H_2_O atmosphere. Twenty-four hours pre-transfection, 1.3 × 10^5^ cells were seeded in a 24-well plate. The next day, either 450 ng of pRL-TK vector (Rluc) and 50 ng of pGL3-control vector (Firefly luciferase reporter, Fluc) or 25 ng of psiCHECK vector (containing both Rluc and Fluc reporter genes) and 475 ng of pUC19 carrier vector were transfected with 0.5 µL of Lipofectamine 2000 (Invitrogen) per well. Twenty-four hours later, the cells were lysed in passive lysis buffer (Promega) and the luciferase assays were performed following the Dual-luciferase Reporter Assay manufacturer's protocol (Promega) using the Glomax 20/20 luminometer. The Rluc value was normalized over the Fluc value. The percentage (%) of luciferase activity was then set to 100% for all of the G-tracks G/A-mutant constructs, while the luciferase activity values of the other constructs were divided by the value of their corresponding G-tracks G/A-mutant and multiplied by 100. The means and standard deviations were calculated from at least two independent experiments. Statistical significance was evaluated with an unpaired Student *t*-test using the GraphPad Prism 6.02 software.

## SUPPLEMENTAL MATERIAL

Supplemental material is available for this article.
